# Investigating mechanisms underlying neurodevelopmental phenotypes of autistic and intellectual disability disorders: a perspective

**DOI:** 10.3389/fnsys.2013.00075

**Published:** 2013-10-31

**Authors:** Tim Kroon, Martijn C. Sierksma, Rhiannon M. Meredith

**Affiliations:** Department of Integrative Neurophysiology, Centre for Neurogenomics and Cognitive Research (CNCR), Neuroscience Campus Amsterdam, VU UniversityAmsterdam, Netherlands

**Keywords:** neurodevelopmental disorders, critical periods, gene expression, phenotype, development

## Abstract

Brain function and behavior undergo significant plasticity and refinement, particularly during specific critical and sensitive periods. In autistic and intellectual disability (ID) neurodevelopmental disorders (NDDs) and their corresponding genetic mouse models, impairments in many neuronal and behavioral phenotypes are temporally regulated and in some cases, transient. However, the links between neurobiological mechanisms governing typically normal brain and behavioral development (referred to also as “neurotypical” development) and timing of NDD impairments are not fully investigated. This perspective highlights temporal patterns of synaptic and neuronal impairment, with a restricted focus on autism and ID types of NDDs. Given the varying known genetic and environmental causes for NDDs, this perspective proposes two strategies for investigation: (1) a focus on neurobiological mechanisms underlying known critical periods in the (typically) normal-developing brain; (2) investigation of spatio-temporal expression profiles of genes implicated in monogenic syndromes throughout affected brain regions. This approach may help explain why many NDDs with differing genetic causes can result in overlapping phenotypes at similar developmental stages and better predict vulnerable periods within these disorders, with implications for both therapeutic rescue and ultimately, prevention.

## Rationale and background

Cognitive disorders, including intellectual disability (ID) and autism spectrum disorders (ASD) are genetically and phenotypically highly heterogeneous. To date, more than 450 candidate genes are associated with ID and many hundreds with ASD—numbers predicted to rise with the routine usage of high throughput sequencing technology (Mitchell, 2011; van Bokhoven, 2011; State and Sestan, 2012). Despite the heterogeneity of genes underlying both syndromic and non-syndromic forms of ID and ASD, they are often characterized by early onset of symptoms, overlapping developmental delays and prominent regression of acquired behaviors in ASD during early childhood (Geschwind and Levitt, 2007). However, the underlying mechanisms and early temporal dysregulation in neuronal signaling pathways that trigger neurodevelopmental disorder (NDD) onset and regulate symptoms are not fully understood.

Many known candidate genes for both ID and ASD are expressed synaptically, regulate synapse function and morphology or are themselves regulated by synaptic activity (Ramakers, 2000; Zoghbi and Bear, 2012). For known monogenic NDD syndromes, genetic mouse models such as the Fragile X mental retardation 1 knockout (Fmr1-KO) mouse for Fragile X syndrome (FXS; Bakker et al., 1994) or tuberous sclerosis protein 1/2 (TSC 1/2) models for tuberous sclerosis (Meikle et al., 2007; Ehninger et al., 2008) have enabled the functional study of these genes in the intact brain. For many such mouse models, the target gene is permanently disrupted early on in development, either globally or in a cell-type specific manner. Nevertheless, recent data reveal developmentally regulated and transient synaptic phenotypes in NDD models despite a permanent alteration in genotype (Meredith et al., 2012).

Here, we propose that key developmental aspects of NDD symptoms can be better understood by focusing on the interactions between synaptic NDD gene pathways and the underlying known critical periods in the neurotypical brain. Further, we propose that clustering NDD gene groups on their neuro-spatio-temporal expression profiles, rather than biological functions alone, may reveal novel NDD genes and explain the developmental regulation of specific symptoms. Combining knowledge of key gene networks dysregulated in NDDs and their role during critical periods may elucidate causal mechanisms for symptom onset and further our understanding of critical periods in neurotypical brain development. The ideas presented are formulated as three testable hypotheses for validation in known genetic NDD syndromes (Box 1).

**Box 1 Hypotheses**

**Hypothesis 1**

Dysregulation of synaptic pathways occurs at the subcortical level in NDDs at ‘presymptomatic’ stages.

**Hypothesis 2**

Dysruption of critical periods in subcortical regions such as brainstem precedes and consequently disrupts critical periods in thalamus and then cortex.

**Hypothesis 3**

No differences in synaptic networks or critical periods in NDDs occur prior to the neurotypical pre- or postnatal expression of the NDD gene in that brain region.

## Developmental delays and postnatal onset in NDDs

Although ASDs and several forms of ID are heterogeneous, symptoms often emerge during early development. Initial symptoms such as hypotonia and developmental delay of motor activities, impaired social interactions, repetitive behaviors and epileptic seizures can manifest early in life (Zoghbi and Bear, 2012). Hypotonia during early neonatal periods is correlated with delayed motor skill development in infancy and characteristic for many monogenic disorders including FXS, Angelman syndrome and syndromic Oligophrenin-1 mutation ID (OPHN1; Kau et al., 2000; Bergmann et al., 2003; Clayton-Smith and Laan, 2003; Williams et al., 2006). Additionally, there is high comorbidity of epilepsy in ID and autism and often, seizure activity is developmentally regulated (Gillberg and Billstedt, 2000; Amiet et al., 2008; Ramamoorthi and Lin, 2011). For OPHN1 ID, absence and myoclonic jerks often develop into seizures with increasing frequency in the first 12 months (Bergmann et al., 2003). In Rett Syndrome, developmentally regulated seizures also occur along with regression of behaviors after 6–18 months of neurotypical progress (Steffenburg et al., 2001; Weaving et al., 2005). In many such disorders, the earlier the onset of first symptoms, the more severe the locomotor dysfunction and impairments in language acquisition (Gratchev et al., 2001). Impairments in speech and social interactions are commonly reported to be delayed in syndromes such as FXS and Angelman, where they may be characterized as core symptoms or as part of an ASD comorbid with ID (Gillberg and Billstedt, 2000; Amiet et al., 2008; Ramamoorthi and Lin, 2011). Altogether, the overlapping symptoms, their temporally restricted onset and an overall developmental delay suggest a common NDD etiology in brain development.

The impact of developmental delays is not just confined to symptom onset but could extend beyond the presentation period to disrupt subsequent developmental stages. This concept of “sleeper effects” is illustrated for permanent visual impairments emerging later on in life due to a lack of early sensory experience (Maurer et al., 2007). Early hypotonia and impaired motor skills, or aberrant sensory modulation and social avoidance are paired examples where earlier developmental impairments can have lasting consequences upon later behavior, despite the fact that the initial impairment was transient or lessened with age (Baranek et al., 2006; Ben-Sasson et al., 2009). Although these reports were not longitudinal, the correlations suggest that impairments of sensory or motor functions affect the acquisition of complex behaviors such as speech, language and social interaction. However, while the prevalence of sensory impairments is significantly greater in those with ID than in the general population (Carvill, 2001) it is important to note that not all pre- or early postnatal sensory impairments such as congenital blindness or deafness are associated with later diagnosis of ID or autistic syndromes. The strong association with sensory impairments may, in part, arise from infections or perinatal events that cause extensive neurological damage but for genetic conditions such as Usher syndrome, specific visual and auditory impairments can occur without cognitive or social disabilities. Regardless of the genetic and environmental heterogeneity in underlying NDDs, impaired development is characteristic for both syndromic and non-syndromic NDDs. Here, within the category of NDDs we focus on genetically identified IDs and ASDs as these disorders are widely studied in humans and investigated in animal models. Further, we speculate that the syndromic and nonsyndromic disorders converge on similar developmentally regulated mechanisms.

## Critical periods and normal brain development

Critical periods are developmental time-windows during which external stimuli have a heightened influence on the proper development of an organism. While the early stages of development are largely based on hard-wired genetic and molecular cues (Chilton, 2006; Marin et al., 2010), at later stages neuronal activity becomes an important factor contributing to circuit development in the brain (Lendvai et al., 2000; Spitzer, 2006). This activity can be intrinsically generated (Golshani et al., 2009; Rochefort et al., 2009) or induced by sensory stimulation (Siegel et al., 2012). Although neuronal circuits remain malleable by external stimuli throughout life, most circuits are especially sensitive to external input during restricted time-windows, or critical periods (Knudsen, 2004; Hensch, 2005). Consequently, disruptions of external input have a much greater effect during the critical period than at other times and these effects can be irreversible. In the primary visual cortex (V1) of the cat, prolonged closure of one eyelid in kittens, shifts V1 neuron responsiveness toward the open eye (Wiesel and Hubel, 1963). This effect is largely absent in adult cats. Since then, this shift in ocular dominance in juvenile mammals has become the most widely studied instance of a critical period. Subsequently, critical periods have been found in many cortical regions and sensory modalities, such as the somatosensory (Fox, 2002) and auditory systems (Barkat et al., 2011; Yang et al., 2012).

 Development is typically a set of processes influencing both behavioral and biological characteristics which occur sequentially (Michel and Tyler, 2005). It is interesting to note that there seems to be a sequential hierarchical structure to the order in which different critical periods occur. In somatosensory cortex, restricted critical periods for thalamocortical and then cortico-cortical synapse connectivity and maturation occur in a regulated layer-specific sequence (Fox, 2002; Feldmeyer et al., 2013). In the visual cortex, layer IV receives subcortical input, which is subsequently processed in both superficial and deep layers. The critical period for ocular dominance in these cortical layers lasts longer than that of inputs to layer IV (Daw et al., 1992). This may explain why there seems to be a lack of clearly defined critical periods for higher order functions involving sensory cortical networks spread across different layers. In the visual condition amblyopia (lazy eye), treatment is most effective in young children, but it can also still be treated in adults (Polat et al., 2004). This phenomenon whereby sensory plasticity underlying acquired behaviors can occur in the adult nervous system, albeit at a less effective level, also applies to the auditory system. For example, in congenitally deaf children, cochlear implants are most effective when treatment starts at an early age. The earlier the implantation, the more likely these children are to develop spoken language (Nicholas and Geers, 2007). Children who receive cochlear implants after the age of seven do not develop normal cortical responses to auditory stimuli (Sharma et al., 2009). However, there is no clear cut-off when cochlear implantation ceases to be useful, as implantation after this age does improve hearing (Harrison et al., 2005). Similarly, although second-language acquisition is most effective when started before the age of 4, adults retain the ability to learn new languages, albeit less fluently (Werker and Tees, 2005). Furthermore, musicians who start musical training before age 7 on average ultimately perform better than those who start training at a later age (Penhune, 2011), but learning to play music is still possible during adulthood. Thus, developmental time-frames for plasticity exist at both the synaptic and behavioral levels within which the greatest periods of phenotypic change occur and where lack of sensory experience has the most significant effects. These timeframes are commonly referred to as “critical” periods when investigating mechanisms of synaptic and molecular changes. They are also referred to as “sensitive” periods for many behaviors, although the distinction of usage and the exact ending of these periods is not always clear-cut (Johnson, 2005; Michel and Tyler, 2005). Here, we use the term “critical period” to refer to both synaptic and behavioral phenotypes that occur during documented neurotypical developmental stages.

 At the level of the synapse, development and formation of functional connections during neurotypical maturation follows an established sequence: initial axonal and dendritic outgrowth, excess formation of immature long thin filopodia-like spines and subsequent pruning of synaptic contacts accompanied by an activity-induced maturation of remaining synapses (Katz and Shatz, 1996; Ethell and Pasquale, 2005; West and Greenberg, 2011). Whilst synapse remodelling is a lifelong process (Holtmaat et al., 2005; Grillo et al., 2013), the peak of synapse development and synaptic connectivity is predominantly established during early postnatal periods in vertebrates (Pan and Gan, 2008). For primary sensory cortices, the network is shaped by sensory input during the critical period coinciding with a high level of synaptic and neuronal remodelling. Thus, during neurotypical development, critical periods for the greatest changes in synaptic circuits in the brain and behavior are defined when the system is most susceptible to change. As such, plasticity of specific phenotypes is heightened relative to earlier or later developmental stages.

## Molecular pathways involved in monogenic NDD converge on synapse function

Aberrant spine morphology is characteristic for individuals with NDDs as post-mortem studies report an abundance of immature, long thin spines and in some cases, altered spine density (Kaufmann and Moser, 2000; Ramakers, 2002; He and Portera-Cailliau, 2013; Maynard and Stein, 2012). Morphological aberrations also occur in non-syndromic ID where dendritic spine impairments correlated with age and severity of developmental disability (Purpura, 1974; Ramakers, 2002). Thus, a body of evidence from human post-mortem studies indicates a strong correlation between altered structural development of synapses and NDDs.

**Figure 1:**
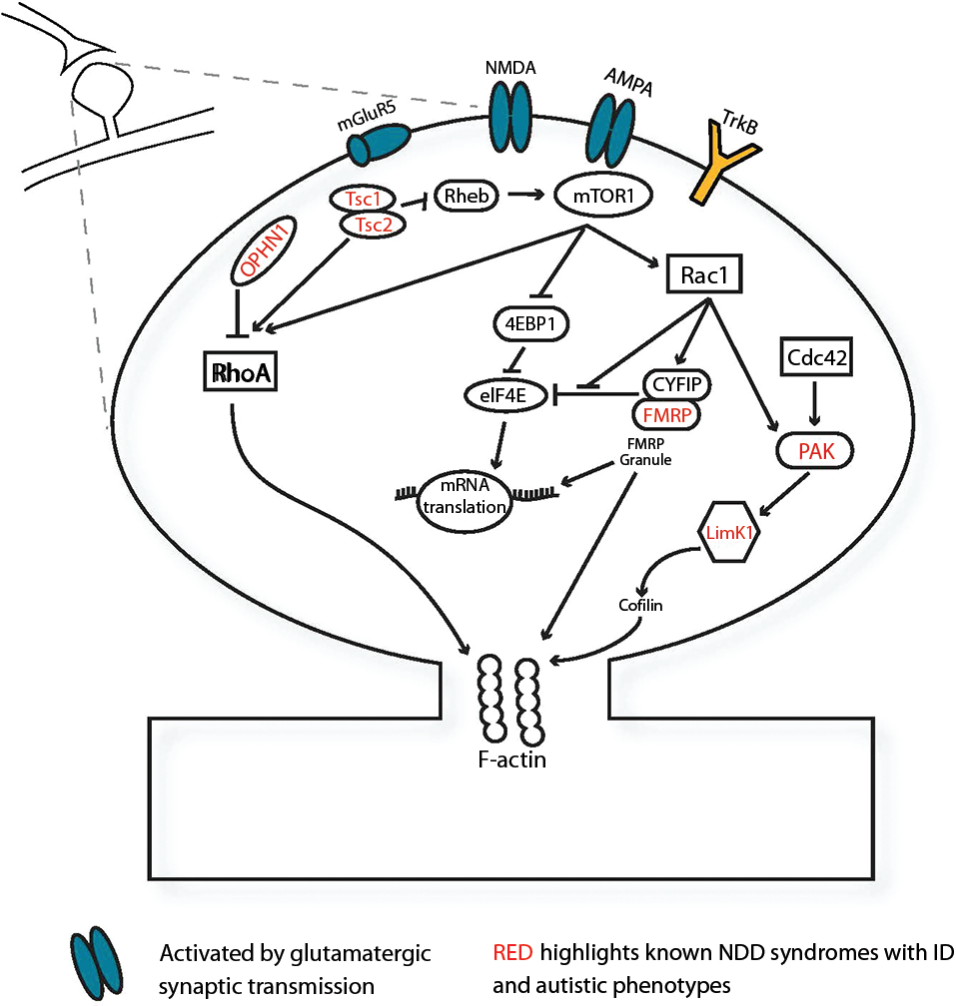
**Several NDD-associated genes function at the synapse**. Monogenic NDD genes (red) expressed in the synapse, illustrated here postsynaptically, mediate spine morphology changes via small GTPase-mediated signaling pathways and F-actin in response to synaptic activation via BDNF and glutamatergic excitation. Abbreviations: **4EBP1**, eukaryotic translation initiation factor 4E-binding protein 1; **AMPA**, 2-amino-3-(3-hydroxy-5-methyl-isoxazol-4-yl) propanoic acid receptor; Cdc42, cell division cycle 42; **CYFIP**, cytoplasmic binding partner of fragile X protein; **eIF4E**, eukaryotic translation initiation factor 4E; **FMRP**, fragile X mental retardation protein; **LimK1**, LIM domain kinase 1; **mGluR5** metabotropic glutamate receptor subunit 5; **mTOR1**, mammalian target of rapamycin 1; **NMDA**, N-Methyl-D-aspartic acid or N-Methyl-D-aspartate Receptor; **OPHN1**, oligophrenin-1; **PAK**, P21-activated kinase; **Rac1**, ras-related C3 botulinum toxin substrate; **Rheb**, Ras homolog enriched in brain; **RhoA**, ras homolog gene family, member A; **TrkB**, TrkB tyrosine kinase; **Tsc 1**, tuberous sclerosis protein 1; **Tsc 2**, tuberous sclerosis protein 2.

Initial stages of synapse formation and neuronal connectivity require modulation of the cytoskeletal F-actin via the Ras homologue subfamily of Rho GTPases. Many genes underlying monogenic NDDs interact directly with Rho signaling protein pathways. (Figure 1; Ramakers, 2002; Ethell and Pasquale, 2005). This family of small-GTPases includes ras homolog gene family, member A (RhoA), ras-related C3 botulinum toxin substrate (Rac1) and cell division cycle 42 (Cdc42), which dynamically regulate protrusion and retraction of spines via cytoskeletal actin remodelling (Tashiro et al., 2000; Ethell and Pasquale, 2005). Small guanosine-5'-triphosphate hydrolyzing enzymes (GTPases) typically cycle between active GTP-bound and inactive guanosine diphosphate- (GDP) bound states. These transitions are dynamically regulated by GTPase activating proteins (GAPs), guanine nucleotide exchange factors (GEFs), and by GDP dissociation inhibitors (GDI) inhibiting the conversion to the active GTP-bound state (Sasaki and Takai, 1998). In syndromic OPHN1 ID, changes in spine morphology are caused by the absence of OPHN1, a RhoA-GAP (Govek et al., 2004). The ID Williams syndrome is linked to the LIM domain kinase 1 (LIMK1) gene, whose product mediates changes in actin and spine morphology via Cdc42 and Rac1 pathways (Edwards et al., 1999). Additionally, LIMK1 interacts with P21-activated kinases (PAKs) which also harbor mutations in many nonsyndromic human ID cases (Allen and Walsh, 1999). Rho family members are activated by extracellular stimuli via growth factors and neurotransmitter release. Brain-derived neurotrophic factor (BDNF), involved in synaptic maturation, activates Rac/RhoA-GEF proteins via TrkB tyrosine kinase (TrkB) receptors and induces spine head growth (Hale et al., 2011). During synaptic activation, glutamatergic transmission activates 2-amino-3-(3-hydroxy-5-methyl-isoxazol-4-yl) propanoic acid receptor (AMPA) and N-Methyl-D-aspartic acid or N-Methyl-D-aspartate (NMDA) receptors and subsequently activates Rho proteins (Sin et al., 2002). Therefore, activity of the Rho proteins is sensitive to synaptic transmission and can regulate activity-induced maturation of the synapse. Synaptic maturation requires structurally modifying the synapse via cell-adhesion proteins including CNTNAP2, neuroligins 3 and 4, neurexin1, δ-catenin and associated Shank and Homer proteins which are frequently implicated in ASD (Tu et al., 1999; Sala et al., 2001; Jamain et al., 2003; Sudhof, 2008; Matter et al., 2009; Anderson et al., 2012). These proteins ensure proper synapse formation by bridging the pre- and postsynaptic sites, acting as a scaffold and stabilizing the cytoskeleton of the synapse (Kosik et al., 2005; Takeichi and Abe, 2005; Penagarikano and Geschwind, 2012). Since the Rho signaling pathways and synapse-spanning complexes are enriched with NDD-related proteins, they provide a direct link between NDDs and aberrant synapse development.

In addition to direct modulation of the cytoskeleton, many NDD-related proteins are regulators of gene transcription, mRNA translation and ultimately protein synthesis (Nan et al., 1997; Bagni and Greenough, 2005; Kelleher and Bear, 2008; Guy et al., 2011). NMDA-dependent, metabotropic glutamate receptor (mGluR)-dependent and BDNF-induced synaptic plasticity mechanisms depend on protein synthesis via the Ras-mitogen-activated protein kinase (Ras-MAPK) pathway and directly or indirectly modulate TSC 1/2 complex activity (Sweatt, 2004; Banko et al., 2006; Gong and Tang, 2006; Kelleher and Bear, 2008). Misregulation of mRNA translation, particularly for synaptic proteins, is proposed to underlie many “synaptopathies” with impairments or loss of fragile X mental retardation protein (FMRP), TSC 1/2, ubiquitin-protein ligase E3A (UBE3A) and eukaryotic translation initiation factor 4E (eiF4E) all causing altered protein synthesis (Auerbach et al., 2011; Zoghbi and Bear, 2012; Santini et al., 2013). Furthermore, altered transcriptional regulation via methyl CpG binding protein 2 (MECP2) is also linked to prominent impairments in Rett syndrome (Guy et al., 2011). Thus, the effects of many NDD-linked genes occur at the level of spine morphology, synapse function and regulation of local protein synthesis in the developing and adult mammalian brain.

## Temporal synaptic phenotypes and critical periods in NDD mouse models

Across different NDD mouse models, studies consistently report an abundance of thin immature filopodia-like spines and small spine heads (Meng et al., 2002; Galvez and Greenough, 2005; Cruz-Martin et al., 2010; Maynard and Stein, 2012; Powell et al., 2012) and/or an altered spine density (Dolen et al., 2007; Meikle et al., 2007; Yashiro et al., 2009; Sato and Stryker, 2010; Powell et al., 2012). In many models, alterations in synaptic phenotypes are often reported at one developmental stage, often corresponding to adult symptomatic stages or a period of 2–3 weeks postnatal age during which extensive refinement and plasticity of synapses occurs in rodent brain. However, data derived from longitudinal studies support the notion of developmentally regulated and transient phenotypes in NDD models.

In typically developing somatosensory cortex, spine morphology changes greatly between postnatal weeks 1–4, shifting from a high proportion of transient, thin “immature” spines to more mature, long-lasting stubby spines (Ethell and Pasquale, 2005). However, in Fmr1-KO mice, this transition is delayed at 2 weeks of age (Cruz-Martin et al., 2010) but both spine morphology and dynamic turnover are normalized around one month of age (Nimchinsky et al., 2001; Cruz-Martin et al., 2010). Intriguingly, the immature spine phenotype reappears in the adult Fmr1-KO mice (Galvez and Greenough, 2005) similar to the pattern of transient changes in spine morphology observed in the down syndrome cell adhesion molecule knockout (DSCAM-KO) mouse model for Down Syndrome (Maynard and Stein, 2012). Critical periods in the somatosensory cortex occur in a sequential pattern, from subcortical to later cortico-cortical changes (Fox, 2002; Feldmeyer et al., 2013). Transient phenotypes are also observed in thalamocortical pathways: in Fmr1-KO mice, enhanced NMDA/AMPA synaptic ratios and altered plasticity occur during the first but not by the end of the second postnatal week, indicating developmental delays within the neurotypical critical period for this pathway (Harlow et al., 2010). In contrast, premature maturation of thalamocortical NMDA/AMPA ratios and plasticity occurs in heterozygous mice for SynGap1, a Ras GTPase-activating protein implicated in ID and ASD but this also normalizes at the end of the first postnatal week (Clement et al., 2013). During the second postnatal week, after the cessation of thalamocortical plasticity, decreased connectivity strength and diffuse axonal branching occurs in cortical circuits between layers 4 and 2/3 of Fmr1-KO mice. Again, these deviations from neurotypical development are restricted and normalize one week later (Bureau et al., 2008). Thus, in somatosensory cortex, many transient changes occur during established critical periods for particular synaptic pathways. Such transient NDD phenotypes are not limited to sensory cortex but also occur in other brain regions including medial prefrontal cortex (Testa-Silva et al., 2012), amygdala (Vislay et al., 2013) and olfactory epithelium (Palmer et al., 2008).

In addition to aberrations in critical periods for synapse and circuit formation, dysregulated synaptic phenotypes occur during critical periods for adaptation to sensory deprivation. Ocular dominance and experience-dependent plasticity mechanisms in response to monocular deprivation (MD) are documented well for the mouse visual cortex and occur during a restricted postnatal period. In Fmr1-KO mice, a short MD period induced a significantly smaller reduction in response in the deprived cortex and an enhanced potentiation of input from the open eye compared to wildtype (WT) mice (Dolen et al., 2007). A lack of plasticity in the deprived cortex after MD was also observed in m-UBE3A-KO mice, a model for Angelman syndrome where the maternal gene copy is lacking (Yashiro et al., 2009; Sato and Stryker, 2010). This effect was not due to a developmental shift in the critical period for m-UBE3A-KO mice since no change in response to MD was observed if the deprivation occurred before, during or after the neurotypical critical period (Sato and Stryker, 2010).

The closure of the critical period for ocular dominance can be manipulated by changes in inhibition or by sensory deprivation through rearing mice in the dark (Hensch, 2005). In heterozygous MECP2-KO female mice, ocular dominance plasticity in response to MD could be induced far beyond the neurotypical critical period into young adulthood, suggestive of a lack of maturation and normal closure of this plasticity mechanism (Tropea et al., 2009). Early synaptic development of the visual system in MECP2 null mice appears normal up to P21 but is followed by later impairments of retinogeniculate synapses (Noutel et al., 2011), increased cortical inhibition and ultimately, impaired visual acuity (Durand et al., 2012). These later developmentally regulated changes in the MECP2 mouse model reflect the protein’s proposed role in synaptic maintenance during adult stages (Guy et al., 2007; Robinson et al., 2012) similar to late postnatal onset of impairments in the Cri-du-Chat mouse model (Matter et al., 2009) but in contrast to other NDD models displaying earlier synaptic phenotypic impairments.

What are the consequences of a dysregulated synaptic phenotype or altered critical period in the developing brain? During retinotopic map development, disruption of synaptic activity during an early critical period alters later neuronal connectivity within the visual system. Desynchronization of early retinal waves of neuronal activity in mouse pups lacking the *β*2- nicotinic acetylcholine receptor subunit is a transient phenotype restricted to the first but not second postnatal week of development. This altered activity results in an impaired finescale refinement of retinal axons in the brainstem (Grubb et al., 2003; Mclaughlin et al., 2003), altered geniculocortical projections (Cang et al., 2005) and a decrease in visual acuity at the cortical level (Rossi et al., 2001). Therefore, disruption or loss of an early critical period can influence both functional and structural connectivity not only in the affected region but in other areas of the sensory processing system and result in altered sensory perception. Applying this principle to NDDs, early or transient alterations in synaptic phenotypes during known critical periods could account for later aberrations in synaptic function, morphology and potentially even behavioral impairments of sensory information processing that characterize many of these disorders.

## Neural connectivity and excitation-inhibition balance in NDDs

Abnormalities in connectivity of excitatory and inhibitory neurons in NDDs are documented at many different levels from whole-brain functional imaging studies to electron microscopic changes in synaptic morphology (Kaufmann and Moser, 2000; Belmonte et al., 2004; Belmonte and Bourgeron, 2006; Dinstein et al., 2011). Dysregulation of excitatory/inhibitory (E/I) balance is proposed to impair neural processing and underlie cognitive deficits in many ID and autistic syndromes (Rubenstein and Merzenich, 2003). E/I is aberrant in many NDD mouse models: some have increased excitability [FXS: (Hays et al., 2011; Testa-Silva et al., 2012; Goncalves et al., 2013), TSC: (Bateup et al., 2013)], ASD models (Peca et al., 2011; Penagarikano et al., 2011; Clement et al., 2012) whilst others show increased inhibition [Downs: (Fernandez et al., 2007; Chakrabarti et al., 2010; Kleschevnikov et al., 2012) Rett: (Dani et al., 2005; Noutel et al., 2011; Durand et al., 2012), but see Calfa et al. (2011) and Kron et al. (2012)]. Thus dysregulation of either excitation or inhibition can disrupt the correct E/I balance in NDDs.

The interaction between E/I balance and development of synaptic networks during critical periods is likely a complex and finely tuned set of processes. In visual cortex, maturation of inhibition triggers critical period onset accompanied by regulation of excitatory synapse strength via activity-dependent mechanisms (Hensch, 2005). Thus both timing and synaptic maturation during critical periods depend upon a delicate interplay of both excitatory and inhibitory transmission and as such, are vulnerable to NDDs affecting E/I balance directly. An indirect effect of NDDs upon E/I balance could also arise if perturbations occur to delay or disrupt a critical period, thereby altering the correct development of synaptic connectivity. Given the sequential nature of synapse development from thalamocortical to sensory cortical regions, an early aberration affecting E/I balance during one critical period could give rise to impairments in a subsequent critical period of a cortical network. This may occur either directly via the same E/I—critical period mechanism or as a consequence of, for example, impairments in the outgrowth of axonal projections from one synaptic network to the next.

A prevailing hypothesis in NDD research proposes a weakening of long-range projections in addition to a strengthening of local-range connectivity in the brain (Belmonte et al., 2004; Just et al., 2004). Local hyperconnectivity of excitatory networks in neocortex is observed in mouse models for FXS (Testa-Silva et al., 2012; Goncalves et al., 2013) and ASD (Rinaldi et al., 2008; Qiu et al., 2011) but Rett syndrome models show local hypoconnectivity (Dani et al., 2005). However, significantly less is known about long-range connectivity at the synaptic level in NDD mouse models or whether developmental trajectories are misregulated. It is likely that impairments in long-range projections in NDDs are not global but rather synapse-specific: alterations in long-range projections occur at cortical but not thalamic inputs to the lateral amygdala in a mouse model for Rett syndrome (Gambino et al., 2010) and in the ID associated gene il1rapl1 mouse model, thalamo-amygdala projections differ only on to principal cells but not interneurons (Houbaert et al., 2013). Furthermore, the period for normal synapse elimination and maturation of long-range projections to lateral amygdala occurred after 3 months of age, indicating that refinement of this synaptic pathway occurs relatively late in postnatal development and could potentially be disrupted by many other early critical period impairments (Gambino et al., 2010). Given the tightly regulated growth of the brain and sequential patterns of development from one synaptic network to another (Ben-Ari and Spitzer, 2010), we propose that long-range connectivity may be particularly vulnerable in NDDs, especially where the NDD-linked genes are strongly expressed at prenatal or early postnatal time-windows in brain development (Meredith et al., 2012). In a recent study, preliminary data reported infants at high risk for ASD had higher long-range functional connectivity than those at low ASD risk at 3 months age but lower connectivity at 12 months (Keehn et al., 2013). Thus longitudinal studies of interregional projections in the brain could reveal whether the key NDD hypothesis of weakened long-range connectivity is specific to the mature brain or applies also to early developmental stages, and how early brain connectivity relates to the onset of NDD symptoms.

## Mechanisms underlying critical periods and NDDs

The existence of sensitive time-windows for the manifestation of symptoms in animal models of neurological and neuropsychiatric disorders has recently been proposed (Leblanc and Fagiolini, 2011; Marco et al., 2011; Martin and Huntsman, 2012; Meredith et al., 2012). Here, we hypothesize that the concept of critical or sensitive periods can be applied to underlying mechanisms of NDDs in two ways.

**Figure 2:**
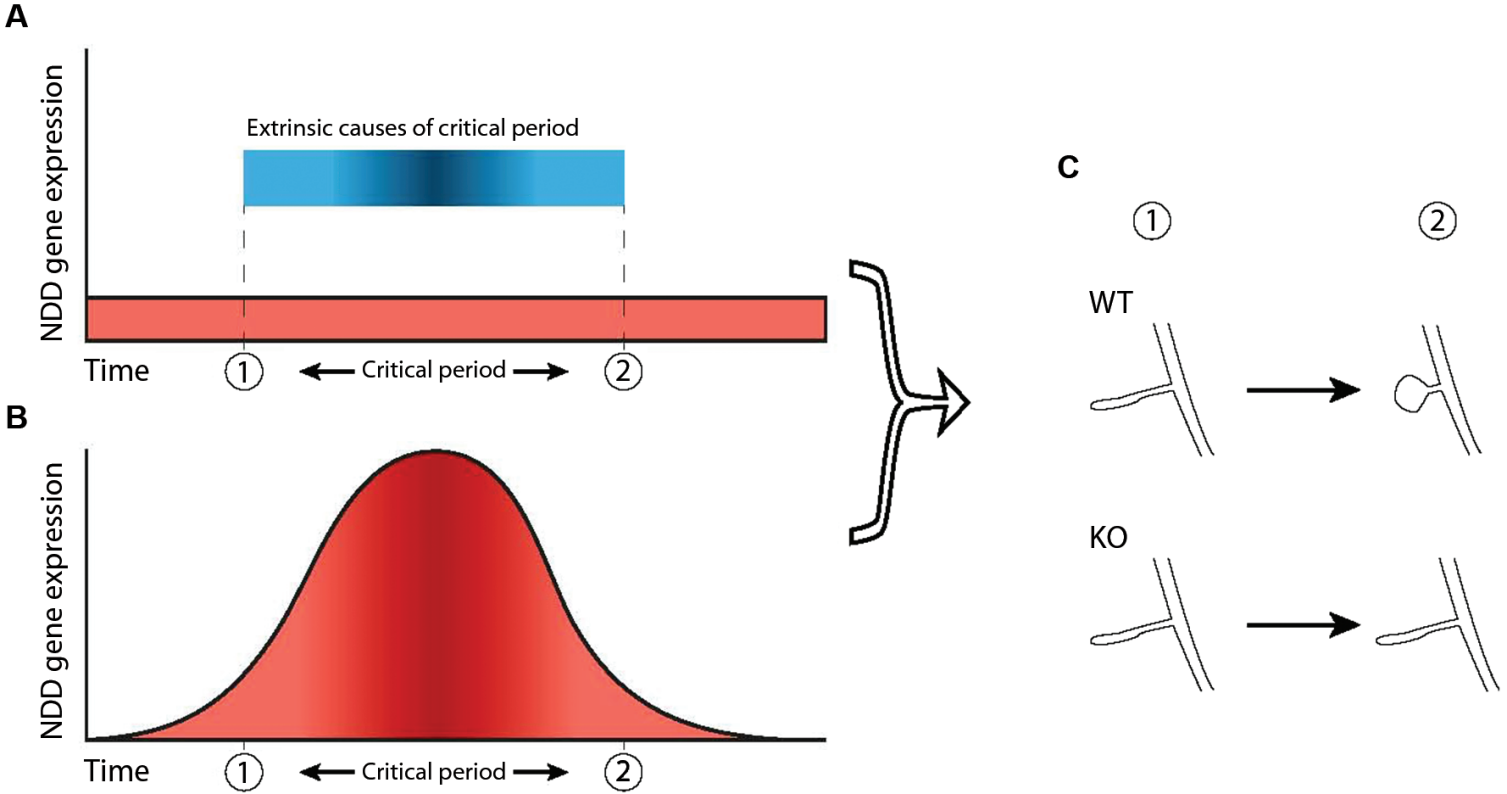
**Two ways in which dysfunction of NDD genes can dysregulate critical periods**. A critical period is shown here as the timeframe between “1” and “2”. **(A)** In this scenario, the critical period is caused by external factors (blue bar) not related to the NDD gene and expression of the NDD gene in wild-type is not necessarily atlered before, during or after the critical period (red area). However, the NDD gene plays a role downstream of these external factors and is necessary for phenotypic change to take place, thereby indirectly regulating not the occurrence but the outcome of the critical period. Hence, dysfunction of the gene leads to an impaired critical period. **(B)** Increased NDD gene expression (red area) directly regulates the critical period and causes it to occur, independent of external factors. Therefore, dysfunction of the NDD gene causes the critical period to be absent completely. **(C)** In both scenarios, the NDD gene is necessary for the phenotypic change that takes place during the critical period (“WT” vs “KO”), represented here by maturation of spine morphology.

First, the underlying pathology of NDDs could arise through aberrant interactions during existing critical period mechanisms that are in place during neurotypical development (Figures 2A, C). For example, both ocular dominance plasticity and mapping of frequency representation during their respective critical periods are impaired in the Fmr1 KO mouse but can be restored by reduction of metabotropic glutamate receptor subunit 5 (mGluR5) expression or pharmacological blockade (Dolen et al., 2007; Kim et al., 2013). The Fmr1 gene product, FMRP, is activated following mGluR5 stimulation and regulates synaptic mRNA translation and (Weiler et al., 1997) mGluR5 activation is necessary for certain types of synaptic plasticity (Huber et al., 2000; Raymond et al., 2000). Attenuation of mGluR5 signaling dysregulates both experience-dependent NMDA receptor expression and synaptic plasticity in young and adult visual cortex, respectively (Tsanov and Manahan-Vaughan, 2009). Therefore, the absence of FMRP in FXS affects the level of synaptic plasticity via mGluR5-mediated signaling dysregulation, which in turn affects the level of response during the critical period for ocular dominance.

The timing aspects of known critical periods in NDDs could also be affected via GABAergic inhibition. GABAergic inhibition is significantly altered in many NDDs (Rubenstein and Merzenich, 2003; Chattopadhyaya and Cristo, 2012). Intact GABAergic inhibition is necessary for the critical period for ocular dominance to occur: KO mice lacking the 65 kD isoform of the GABA production protein glutamate decarboxylase (GAD65) have impaired GABA function and do not show a normal critical period for ocular dominance (Hensch et al., 1998). The critical period can be induced experimentally by pharmacologically increasing GABA*_A_* receptor function (Hensch et al., 1998; Fagiolini et al., 2004). This opening of the critical period can be achieved independently of the age of the mice, indicating that adequate GABAergic signaling is necessary for the critical period to occur, while other mechanisms that act during the critical period are already in place. Thus, an alteration in GABAergic inhibition during brain development in NDDs can thereby lead indirectly to perturbations in the timing of critical periods.

The second concept to link NDDs and critical periods during development is that the expression profile of the gene underlying an NDD may in itself constitute a critical period during which the effects of the NDD are manifest (Figure 2B). This deviates slightly from the general definition of a critical period, as it does not necessarily pertain to external stimuli affecting network development. In this model, upregulation of a gene at a particular time is necessary for the network to develop normally. It is therefore a critical period in the sense that expression of the gene is necessary during a particular time-frame. This has been shown in a *Drosophila* model for FXS, where reintroduction of the *Drosophila* homologue of FMRP (dFMRP) in the knock-out model rescues certain aspects of synaptic morphology only during a 2 day time-window, but not during earlier development or later in the adult (Gatto and Broadie, 2009).

## Temporally dysregulated gene expression underlying neurodevelopmental brain disorders

Gene expression is a dynamic process throughout life and is tightly regulated on both spatial and temporal dimensions. The transcriptome, the collective expression of multiple genes, differs significantly in a tissue-specific and brain region-specific pattern across both cortical and subcortical structures in mammals (Allen Brain Atlas,^1^ Hawrylycz et al., 2012). Transcriptomic profiles reveal distinct layer-specific and non-layer-specific expression patterns for many thousands of genes in the sensory neocortex of adult mouse (Belgard et al., 2011). Similarly, robust genetic signatures for individual cortical layers and also specific brain regions are found in both human and non-human primates, with greater similarity in lamination between primate species than to rodents (Belgard et al., 2011; Bernard et al., 2012).

Given the protracted development of human brain over many years, it is not surprising that the spatial transcriptome varies considerably over time: in humans, more than 90% of detected genes in the brain are differentially regulated in a spatio-temporal manner from embryonic through to geriatric periods (Kang et al., 2011). The greatest changes in regional gene expression occur during prenatal and early postnatal periods (Colantuoni et al., 2011; Kang et al., 2011). In the mouse brain, cohorts of genes are differentially expressed in the subplate at specific developmental stages from late embryonic through to early and late postnatal periods (Hoerder-Suabedissen et al., 2013). Thus, the transcriptome is tightly regulated in the neurotypical mammalian brain and reveals both restricted expression windows and developmentally changing gradients of gene expression.

**Figure 3:**
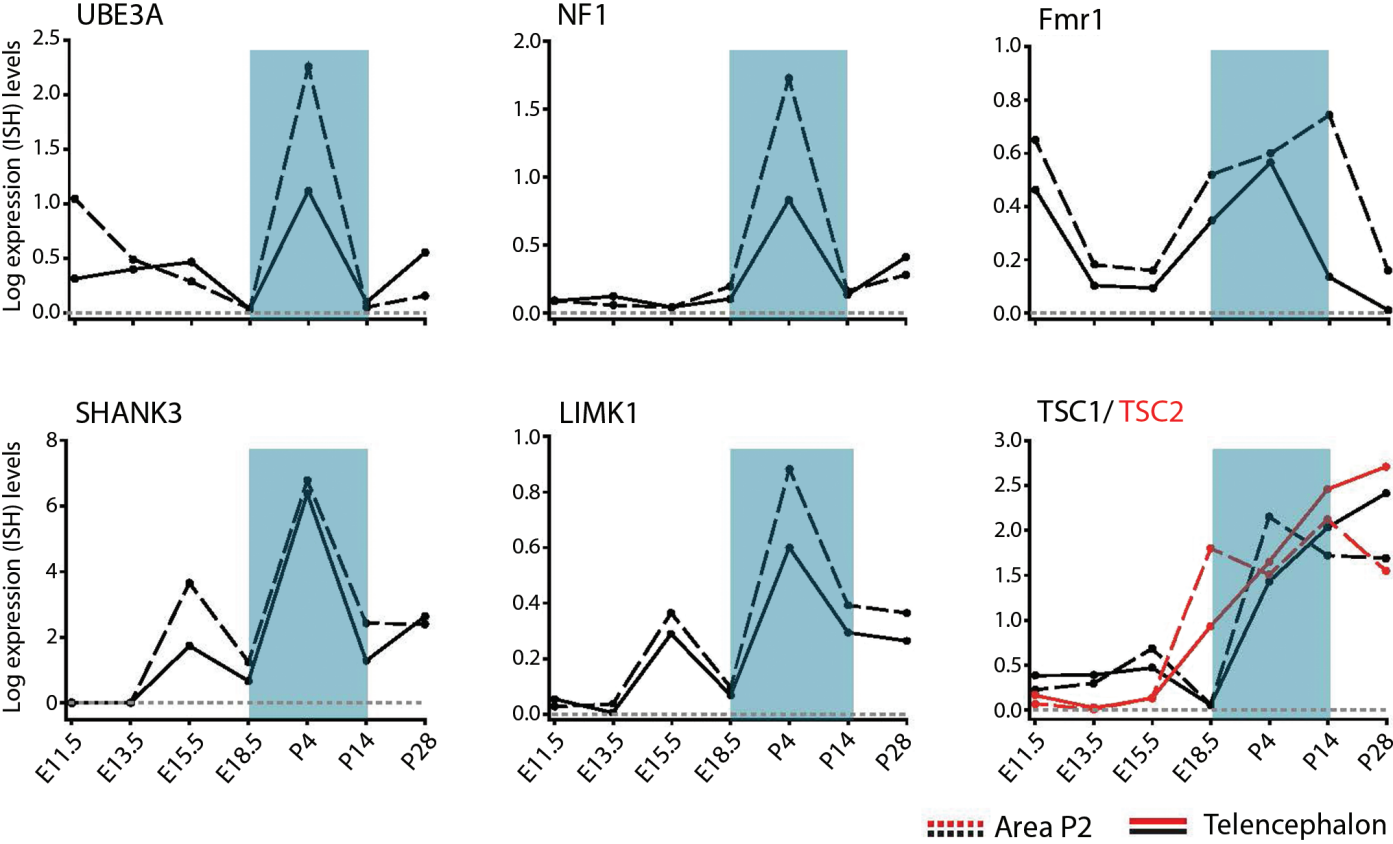
**Temporal regulation of genes for syndromic NDDs during pre- and postnatal brain development**. Developmental profiles of RNA levels for specific monogenic ID and ASD genes in pre- and postnatal development, in telencephalon and thalamic (Area P2) regions. Data extracted from Allen Developing Mouse Brain Atlas Website: ©2012 Allen Institute for Brain Science. Allen Developing Mouse Brain Atlas [Internet].^3^ Abbreviations: **Fmr1**, Fragile X mental retardation 1; **LimK1**, LIM domain kinase 1; **NF1**, neurofibromin; **SHANK3**, SH3 and multiple ankyrin repeat domains 3; **TSC1/TSC2**, tuberous sclerosis protein 1/2; **UBE3A**, ubiquitin-protein ligase E3A.

The developmental regulation of spatial patterns of individual gene expression in the neurotypical brain includes many known NDD candidate genes for monogenic syndromes (Allen Brain Developing Human and Mouse Brain Atlas,^2^). Of interest, many genes linked to ASD show dynamic changes in expression in subplate layers of the mouse cortex, suggesting disruption of early developmentally regulated NDD candidates (Hoerder-Suabedissen et al., 2013). However, the direct functional effects of these gene changes are not yet known. Prominent genes underlying ID and ASD, including Fmr1, neurofibromin (NF1) and TSC 1/ 2 show strong developmental mRNA upregulation particularly from late embryonic stages onwards (Figure 3). For Fmr1, this upregulation is transient, peaking between postnatal days (P) 4 and 14 in telencephalic and thalamic defined regions before decreasing by P28 (Figure 3). Given that transient phenotypes in thalamocortical and cortico-cortical synaptic pathways occur in the Fmr1-KO mouse model, it is plausible that these temporal impairments only arise during periods of peak expression for the Fmr1 gene. That is to say, irregularities in an NDD only result in a phenotype at the time when the NDD gene peak expression would usually occur in neurotypical development. No synaptic NDD phenotype is observed if the gene is not prominently being expressed in that brain region and as such, there is no noticeable impairment in the KO mouse model at that stage.

Exome sequencing of many hundreds of families with individuals affected by ID and ASD reveal a high genetic heterogeneity and many *de novo* mutations (Neale et al., 2012; O’Roak et al., 2012; Sanders et al., 2012). Whilst changes in individual gene expression can be tracked throughout development of the brain, much insight can be gained from groupings of genes based on cell-type expression, synaptic location, similar cellular functions, or spatio-temporal expression patterns (Ruano et al., 2010; Kang et al., 2011; Hawrylycz et al., 2012; Lips et al., 2012). Clustering genes into such modules proves extremely useful for genetically heterogeneous disorders, such as ASD and ID, where individual genes explain, at best, a few percent of cases (Manolio et al., 2009; Ruano et al., 2010; Voineagu et al., 2011). In autistic brain samples, grouping many genes in network modules based on differential expression patterns revealed a downregulation in specific networks related to synaptic function. Additionally, gene networks for astrocytic/microglia function and immune function were enriched relative to neurotypical age-matched brain (Voineagu et al., 2011).

Many NDD gene products regulate expression of many other target genes and orchestrate a cascade of signaling proteins. In FXS, FMRP regulates over 800 mRNA targets (Brown et al., 2001; Vanderklish and Edelman, 2005; Darnell et al., 2011) and alters expression of many different synaptic proteins (Adusei et al., 2010; Klemmer et al., 2011). These FMRP targets are common to regulation throughout the nervous system (Ascano et al., 2012), occur both pre- and postsynaptically and can be grouped according to broad biological functions (Darnell et al., 2011). Thus, for complex disorders, a gene clustering approach on differential expression patterns may likely yield many new targets and therefore insights into the mechanistic basis of these NDD syndromes.

To-date, much emphasis is placed upon the individual signaling pathways dysregulated in specific monogenic NDDs. However, it is apparent that there may be key “hubs” that act as common points of dysregulation within the many signaling pathways in ID and ASD (Bill and Geschwind, 2009; Sakai et al., 2011; Voineagu et al., 2011; Zoghbi and Bear, 2012). Shared pathophysiological signaling pathways are of importance for rescue strategies of synaptic function, protein synthesis and behavioral impairments in mouse models of FXS, TSC and neuroligin-3 (Auerbach et al., 2011; Baudouin et al., 2012). The heterogeneity of NDDs of ID and ASDs proves a major source of difficulty for both researchers and the pharmaceutical industry to propose unifying mechanisms that underlie these disorders and importantly, to find viable therapeutic targets. Clinical testing of multiple targets specific for each syndrome is costly both in time and money. Identification of “hub” NDD genes or their key targets with high expression relatively early in development could provide a new therapeutic angle to intervene in particular NDDs. This approach is by no means straightforward and given the sequential development of critical periods in different brain regions, would be difficult to restrict therapeutic actions to specific synaptic pathways. However, the current testing of mGluR5 inverse agonists in phase II and III clinical trials for cognitive and behavioral phenotypes in FXS is being extended to younger children (Levenga et al., 2010,^4^). Whether developmental age in clinical trials affects outcome is not known, but in the Fmr1-KO mouse model, a greater effect of mGluR5 blockade was observed upon rescue of spine morphology in young compared to old neurons (Su et al., 2011). Furthermore, these findings will have implications for other NDDs with potential for early developmental dysregulation of mGluR5 signaling (Zoghbi and Bear, 2012).

## Testable hypotheses for validation in NDDs

On these bases outlined, we propose three testable hypotheses (Box 1) to guide further investigation into neurobiological mechanisms for pathology of NDDs:

During development of sensory systems in the neurotypical brain, critical periods occur in a sequential pattern from brainstem, to thalamus to cortical regions as synapses form, refine and mature. Given that critical periods at thalamocortical and cortico-cortical synaptic pathways are affected in NDDs, we propose that dysregulation of synaptic pathways occurs at the subcortical level in NDDs at earlier stages than are currently known, during “presymptomatic” stages (**Hypothesis 1**). For human NDD syndromes, this could point towards prenatal and early neonatal changes in brain formation and function at stages not currently tested in the clinic. The implications of abnormalities in brain activity at such early developmental stages would be significant initially for detection and screening for NDDs in the fetus or newborn baby and raise possibilities for therapeutic interventions, technological challenges notwithstanding. It may also challenge the notion at which point a child is considered to be presymptomatic, if changes in brain activity are found at increasingly younger developmental stages.

Many NDD genes exhibit prominent expression in subcortical brain regions as well as in more commonly studied cortical circuitry (Allen Brain Atlas^5^). Building on the observations of sequential disrupted critical periods in NDDs, we postulate that in sensory circuits of a NDD, dysregulation of a critical period in subcortical regions such as the brainstem precedes and consequentially disrupts subsequent critical periods in thalamus and then cortex (**Hypothesis 2**). Thus, dysregulation and potential developmental delay for one known critical period would have a knock-on effect for synaptic circuits regulated at later timepoints at downstream synaptic pathways. Little is known regarding subcortical brain regions in NDD mouse models. However, alteration of GABAergic transmission and reduction of GABA-A receptor subunits is reported at postnatal day 7 in ventrolateral brainstem of MECP2 KO mice (Medrihan et al., 2008). Current use of constitutive knock-out mouse models for genetic NDDs are valid experimental tools to detect such early changes: however, conditional knockout models where gene expression can be temporally controlled in specific cell types would better enable proof of a causal relation between a disrupted critical period in subcortical regions directly leading to later cortical impairments. Combining knowledge of the critical periods for specific mouse brain regions in neurotypical normal development with the temporal expression profile of genes implicated in NDDs can guide the spatial and temporal parameters for designing these experiments.

Observations in mouse models of genetic NDD syndromes, demonstrate that alterations in synaptic networks occur during early brain development. Taking the Fmr1-KO mouse model, for example, reported thalamocortical and cortico-cortical synaptic impairments correlate with FMRP expression that occurs in the normally developing brain (Harlow et al., 2010; Meredith et al., 2012). Although it may be purely coincidental that synaptic impairments in an NDD model co-occur with the normal time period for peak expression of that NDD gene, we believe these are directly linked and that the most prominent phenotypic impairments first occur during the period when the gene would be normally activated and most strongly expressed in the brain. Therefore, we propose that no differences in synaptic networks or critical periods in NDDs occur prior to the neurotypical pre-or postnatal expression of the NDD gene in that brain region (**Hypothesis 3**). Thus, a gene with limited postnatal expression in the brain would not give rise to aberrant prenatal synaptic phenotypes since the gene is not normally activated in cells prior to birth. One upshot of this idea is that discovery of prenatal expression patterns of a gene implicated in NDDs may not only lead to detection of prenatal synaptic phenotypes but highlight additional previously unknown functions of a gene during early developmental stages of the nervous system.

## Compensatory mechanisms in synaptic networks and behavioral processing

Alterations in activity levels during early neuronal network development lead to remodelling and compensatory changes in synaptic strength, a phenomenon known as homeostatic plasticity (Turrigiano and Nelson, 2004). This plasticity mechanism enables a network to regulate its synaptic activity in response to the dynamics of the local environment changed by both intrinsic factors and external stimuli, such as sensory input during early postnatal periods (Marder and Goaillard, 2006). Lack or loss-of-function mutations in MeCP2 disrupts homeostatic network plasticity in both developing cortex (Blackman et al., 2012) and hippocampal cultures (Qiu et al., 2012). Further, lack of FMRP disrupts one specific type of homeostatic plasticity dependent upon retinoic acid and protein synthesis in developing hippocampal networks (Soden and Chen, 2010). Thus, later symptomatic changes in brain networks in some NDDs could arise indirectly from impairments in network homeostasis rather than direct synaptic effects of the NDD protein itself.

The transience of synaptic impairments observed during sensitive time-windows (Meredith et al., 2012) could also be influenced by network compensation mechanisms acting to normalize synaptic phenotypes through homeostatic plasticity at that particular developmental stage. For many NDD target proteins, while they may play a key “hub” role in regulating transcription and translation processes in the cell or signaling at the synapse (Bill and Geschwind, 2009; Zoghbi and Bear, 2012), they are not the sole regulator and residual function is likely to be mediated by additional candidates within a synaptic network. Indeed, the initial delays but not absences of key synaptic phenotypes observed in many NDDs (referred against the already known “developmental checkpoints”, Ben-Ari and Spitzer, 2010) could be due to the extra time necessary for compensatory mechanisms to regulate and support the network, taking over residual functions not provided by the (missing) NDD gene.

Compensatory mechanisms may also operate during developmental stages of NDDs at the level of systems processing and behavior (Johnson, 2012). In an imaging study of young children with diagnosed ASD, fMRI revealed significant differences in brain activation patterns compared with neurotypical age-matched children during a simple motion perception task (Kaiser et al., 2010). However, more interestingly, the unaffected siblings of ASD participants with shared genes and an increased risk for later developing ASD showed significantly different activation patterns to both their siblings and neurotypical controls during the task. Increased activation occurred in ventromedial prefrontal cortex and right posterior superior temporal sulcus, two regions associated with motion processing and general executive function skills (Bechara et al., 2000). These neuro-“endophenotypes”, characteristics reflecting susceptibility for a genetic disorder not manifesting as a clinically defined phenotype, could reflect compensatory processing in the brains of those individuals with higher genetic risks for NDDs but not sufficient alterations to warrant a diagnosis.

In conclusion, establishing the mechanisms that underlie early time windows for aberrations in synaptic circuits and impaired behavioral development in NDDs has the potential to reveal new approaches for pharmacotherapeutic correction of brain activity during early development or even new neurobiological gene targets (Levenga et al., 2010; Meredith et al., 2012). Furthermore, we believe this approach outlined in a set of testable hypotheses may reveal dysregulation of brain activity and neuronal circuit formation at significantly earlier presymptomatic stages in nervous system development than previously thought in both syndromic and nonsyndromic neurodevelopmental brain disorders.

## Conflict of interest statement

The authors declare that the research was conducted in the absence of any commercial or financial relationships that could be construed as a potential conflict of interest.

## Footnotes

^1^www.brain-map.org

^2^https://molnar.dpag.ox.ac.uk/subplate

^3^http://developingmouse.brain-map.org

^4^www.fraxa.org

^5^http://www.brain-map.org/
